# Limited Clinical Impact of Genetic Associations between Celiac Disease and Type 2 Inflammatory Diseases: Insights from Mendelian Randomization

**DOI:** 10.3390/biomedicines12071429

**Published:** 2024-06-27

**Authors:** Mahmud Omar, Mohammad Omar, Salih Nassar, Adi Lahat, Kassem Sharif

**Affiliations:** 1 Sheba Medical Center, Faculty of Medicine, Tel-Aviv University, Tel Aviv 6997801, Israel; 2 School of Medicine, V.N. Karazin Kharkiv National University, 61077 Kharkiv, Ukraine; mohammed.nasif8@gmail.com; 3 Edith Wolfson Medical Center, Holon 5822012, Israel; snassar554@gmail.com; 4 Department of Gastroenterology, Sheba Medical Center, Tel-Hashomer 5262100, Israel; adi.lahat@sheba.health.gov.il (A.L.); kassemsharif@gmail.com (K.S.); 5 Department of Medicine B, Zabludowicz Center for Autoimmune Diseases, Sheba Medical Center, Tel-Hashomer 5262100, Israel

**Keywords:** celiac disease, type 2 inflammatory diseases, mendelian randomization, genetic associations, asthma, allergic rhinitis, atopic dermatitis

## Abstract

**Background:** Celiac disease, a gluten-triggered autoimmune disorder, is known for its systemic inflammatory effects. Its genetic associations with type 2 inflammatory diseases like asthma, allergic rhinitis, and atopic dermatitis remain unclear, prompting this study to explore their potential genetic interplay. **Methods:** Utilizing two-sample Mendelian randomization (TSMR), we examined the genetic associations using 15 genetic instruments from GWAS datasets. Our analysis focused on celiac disease and its relation to asthma, allergic rhinitis, atopic dermatitis, and IgE-mediated food allergies. A power analysis was conducted to determine the study’s detection capabilities, and odds ratios (ORs) with 95% confidence intervals (CIs) were calculated using various MR methods. **Results:** Our Mendelian randomization analysis identified statistically significant genetic associations between celiac disease and several type 2 inflammatory diseases, although these were practically insignificant. Specifically, celiac disease was associated with a slight increase in the risk of atopic dermatitis (OR = 1.037) and a minor protective effect against asthma (OR = 0.97). The link with allergic rhinitis was statistically detectable (OR = 1.002) but practically negligible. Despite robust statistical confirmation through various sensitivity analyses, all observed effects remained within the range of practical equivalence (ROPE). **Conclusions:** Our study identifies potential genetic associations between celiac disease and certain type 2 inflammatory diseases. However, these associations, predominantly within the ROPE range, suggest only limited clinical implications. These findings highlight the need for cautious interpretation and indicate that further exploration for clinical applications may not be warranted at this stage.

## 1. Introduction

Celiac disease (CD) is an autoimmune disorder activated by gluten in genetically predisposed individuals [1,2]. It is characterized by inflammation in the small intestine, which can result in the malabsorption of nutrients [2]. It is associated with systemic inflammatory responses that resemble features seen in type 2 inflammatory diseases, such as asthma, allergic rhinitis, and atopic dermatitis [3]. These conditions share chronic inflammatory processes and dysregulated immune responses to environmental triggers [4,5]. 

The genetic landscape of CD includes HLA-DQ alleles, notably DQ2 and DQ8, essential for disease pathogenesis and also implicated in various immune-mediated diseases [6,7]. This genetic link extends to Selective IgA Deficiency (SIgAD), the most common primary immunodeficiency, which frequently co-occurs with CD [8]. Patients with SIgAD often exhibit allergic manifestations, and studies have highlighted a genetic connection between SIgAD and CD, particularly through the HLA-DQB1*02 allele [9,10]. This overlap suggests that SIgAD might provide additional insights into the genetic interplay between CD and Th2-mediated allergic diseases [11]. 

Further, both CD and type 2 inflammatory diseases involve T-helper cell type 2 (Th2)-mediated pathways, critical for allergic responses and some systemic effects of CD [12]. In addition, research indicates the coexistence of Th1/Th17 and Th2 immune responses in CD [13,14], with findings suggesting the elevated incidence of asthma and other immune-mediated diseases in CD patients regardless of their adherence to a gluten-free diet [15]. Such data highlight the complex interplay of immune responses, significantly more prevalent in CD patients compared to the general population [14], and previous research has often been limited to observational studies, which cannot determine directional causality [16,17].

Two-sample Mendelian randomization (TSMR) is an analytical approach that uses genetic variants as instruments to estimate the causal effect of an exposure on an outcome. This method has the power to mitigate confounding and reverse causation, two common issues in observational studies, by leveraging the random allocation of alleles at conception. Thus, TSMR can provide more reliable evidence of a causal association than traditional epidemiological studies [18].

To address these complex interactions, our study employs TSMR to explore the genetic associations between CD and type 2 inflammatory diseases. We aim to determine whether these genetic links uncover clinically relevant pathways that could enhance the diagnostic and therapeutic strategies. 

## 2. Methods

### 2.1. Data Sources

Our study sourced genome-wide association study (GWAS) datasets for celiac disease, asthma, allergic rhinitis, IgE-mediated food allergies, and atopic dermatitis from the MRBase platform. These case–control studies focused primarily on individuals of European ancestry to ensure a genetically homogeneous sample for effective statistical analysis.

### 2.2. Instrumental Variable Selection

Instrumental variables (IVs) were selected from single-nucleotide polymorphisms (SNPs) that reached genome-wide significance (*p* < 5.0 × 10^−8^). To minimize bias due to linkage disequilibrium (LD), SNPs were clumped with an r^2^ < 0.001 within a 10,000 kb window. The selected IVs from the GWAS datasets for each health outcome were rigorously documented, detailing the effect alleles, betas, standard errors, and *p*-values. The strength of each IV was quantified by the F-statistic, with values exceeding 10 signifying adequate instrument strength.

### 2.3. Research Design Assumptions

**Figure 1:**
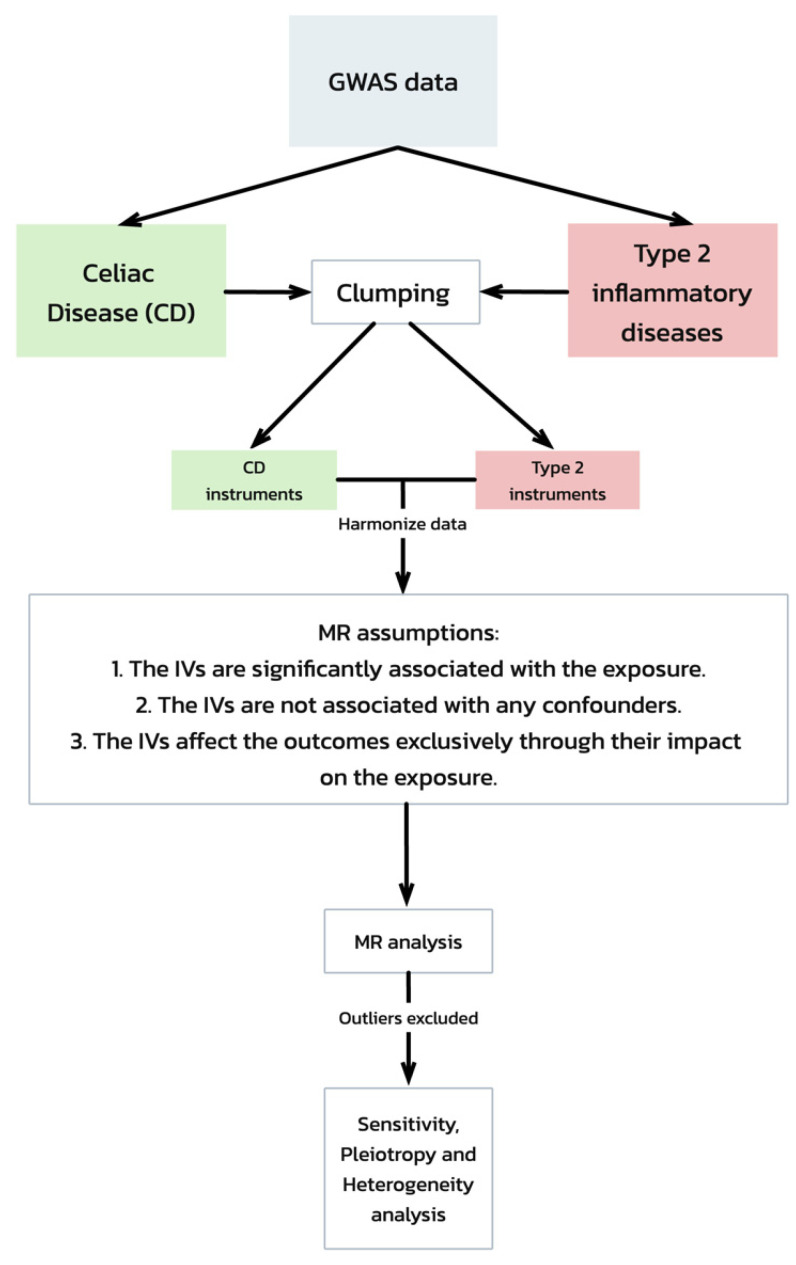
Mendelian randomization (MR) analysis workflow and assumptions. This flowchart depicts the workflow of a Mendelian randomization (MR) analysis evaluating the genetic associations between celiac disease and type 2 inflammatory diseases, starting with GWAS data. It includes steps from data clumping and harmonization to MR analysis, emphasizing the key assumptions required for the validation of the causal inferences. The process concludes with sensitivity, pleiotropy, and heterogeneity tests to ensure the robustness of the results.

Our Mendelian randomization (MR) analysis was predicated on three critical assumptions [19] (Figure 1):

1. The IVs are significantly associated with the exposure (celiac disease).
2. The IVs are not associated with any confounders of the exposure–outcome relationship.
3. The IVs affect the outcomes exclusively through their impact on the exposure.

### 2.4. Statistical Analysis

In our Mendelian randomization (MR) analysis, we harmonized the SNP effects on celiac disease and type 2 inflammatory diseases using MRBase, ensuring allele consistency. Our two-sample MR approach encompassed multiple methodologies: the primary inverse variance weighted (IVW) method integrated SNP data to estimate causal effects, while the weighted median, MR Egger, simple mode, and weighted mode analyses provided supplementary insights, including checks for horizontal pleiotropy. The sensitivity and robustness of our results were verified through leave-one-out and MR Steiger tests, the latter confirming the temporality of the genetic relationship. Incorporating the MR-PRESSO tool allowed us to detect and adjust for outliers. Power calculations assessed our sample’s sufficiency, as outlined by Brion et al. [20] (available at https://shiny.cnsgenomics.com/mRnd/, accessed on 29 February 2024). All statistical procedures were performed via the MRBase web application [21] and R (Version 2023.03.0+386), utilizing the TwoSampleMR and MR-PRESSO packages, using an alpha of 0.05 to define statistical significance. We integrated the region of practical equivalence (ROPE) into our analysis to assess the practical significance alongside the statistical significance. The ROPE thresholds were set at an OR between 0.835 and 1.197, based on the standardized effect size criteria recommended by Kruschke et al. (2018) [22]. This analysis aimed to identify effects within this range as practically negligible, providing a clearer distinction between clinically meaningful and trivial findings.

## 3. Results

### 3.1. Genetic Instrumentation and Sample Characteristics

In our Mendelian randomization (MR) analysis, we used 15 genetic instruments from GWAS data to investigate the associations between celiac disease (ID: ieu-a-1058, sample size: 24,267) and three type 2 inflammatory diseases: atopic dermatitis, asthma, and allergic rhinitis and IgE-mediated food allergied. The GWAS dataset for atopic dermatitis (ID: ebi-a-GCST90027161, sample size: 796,661) included 16,121,213 SNPs, reported by Sliz E et al., 2021 [23]. The asthma (ID: ebi-a-GCST90018795, sample size: 449,500) and allergic rhinitis and IgE-mediated food allergy (ID: ebi-a-GCST90038664, sample size: 484,598, ID: ebi-a-GCST90018625, sample size: 169,716, respectively) datasets were obtained from the studies by Sakaue S et al., 2021 [24] and Dönertaş HM et al., 2021 [25], respectively. 

### 3.2. Association Analyses

#### 3.2.1. Celiac Disease and Atopic Dermatitis

**Figure 2:**
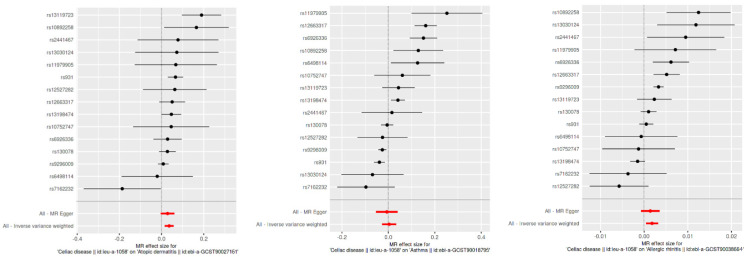
Scatter plot of SNP effects via MR Egger and inverse variance weighted analyses.

**Figure 3:**
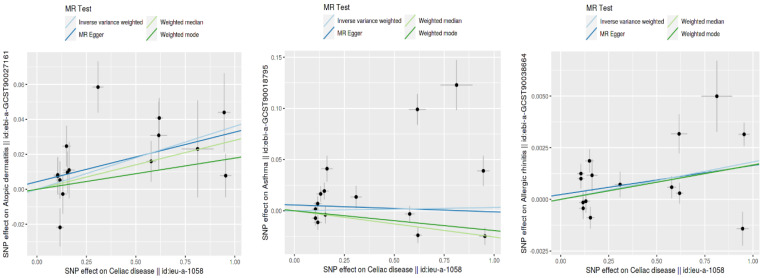
Comparison of SNP effect sizes across different MR methods.

Using the inverse variance weighted (IVW) method, we identified a statistically significant positive genetic association between celiac disease and atopic dermatitis, with an OR of 1.037 (95% CI: 1.015–1.059). This suggests a modest increase in the risk of atopic dermatitis associated with celiac disease. However, when considering the ROPE range of [−0.18, 0.18] for log odds ratios, the practical significance of this effect is called into question, as the effect size does not exceed the ROPE thresholds. The MR Egger intercept showed no evidence of pleiotropy (*p* = 0.545), with moderate heterogeneity observed (Q = 27.58, *p* = 0.016). An additional MR-PRESSO analysis corroborated the initial findings, although adjustments for the identified outliers (indices 8 and 14) did not significantly alter the causal estimates (distortion test *p* = 0.246) (Figure 2 and Figure 3). 

This figure displays the effects of individual SNPs on CD and three type 2 inflammatory diseases: atopic dermatitis, asthma, and allergic rhinitis. Each plot shows the estimated effect sizes and 95% confidence intervals for each SNP, with MR Egger and IVW analyses. The red diamond represents the overall effect estimate using IVW. The figure highlights the consistency and variability in the SNP effects across different analyses.

This figure shows the SNP effect sizes on CD and three type 2 inflammatory diseases using different MR methods: inverse variance weighted (IVW), MR Egger, the weighted median, and the weighted mode. Each plot illustrates the relationship between the SNP effects on CD (x-axis) and those on atopic dermatitis, asthma, and allergic rhinitis (y-axis). The lines represent the estimated effects for each MR method. This comparison highlights the consistency and differences in the effect size estimates across the various methods.

#### 3.2.2. Celiac Disease and Asthma

Our MR analysis suggested a slightly protective effect of celiac disease against asthma, indicated by an OR of 0.97 (95% CI: 0.96–0.98), using the weighted median method. Despite the statistical significance, the effect size lies within the established ROPE range, indicating that this association may lack practical relevance. The MR Egger analysis found no significant association (β = −0.0067, *p* = 0.785), and substantial heterogeneity was present (Q = 120.26, *p* < 0.001). The MR-PRESSO analysis showed a non-significant causal estimate, confirming the minimal practical impact of the findings (β = 0.0032, *p* = 0.840) (Figure 2 and Figure 3). 

#### 3.2.3. Celiac Disease and Allergic Rhinitis

We observed a very small yet statistically significant association between celiac disease and allergic rhinitis, with an OR of 1.002 (95% CI: 1.0004–1.0032), via the IVW method. Despite the statistical significance, the effect size is well within the ROPE range, suggesting negligible practical significance. Notable heterogeneity was present (Q = 57.33, *p* < 0.001), with no pleiotropy detected by the MR Egger intercept (*p* = 0.627). The robustness of these findings was further supported by the MR-PRESSO analysis, although the distortion test indicated a minimal impact from outliers (*p* = 0.715) (Figure 2 and Figure 3). 

#### 3.2.4. CD and IgE-Mediated Food Allergy

The GWAS database ID ebi-a-GCST90018625 was used, which includes individuals with various food allergies but does not specify the types due to database limitations, and no significant correlation was found. The OR was close to 1 (OR = 1.006, *p*-value = 0.745). Confirmatory tests using the MR Egger, weighted median, and IVW methods supported the lack of association (MR Egger OR = 0.993, *p*-value = 0.808; weighted median OR = 1.004, *p*-value = 0.846; IVW OR = 1.006, *p*-value = 0.745). The heterogeneity tests and MR-PRESSO analysis showed no significant pleiotropy or heterogeneity (Egger intercept = 0.008, *p*-value = 0.537), reinforcing the consistency of these findings across different statistical methods.

### 3.3. Causal Directionality Testing

**Figure 4:**
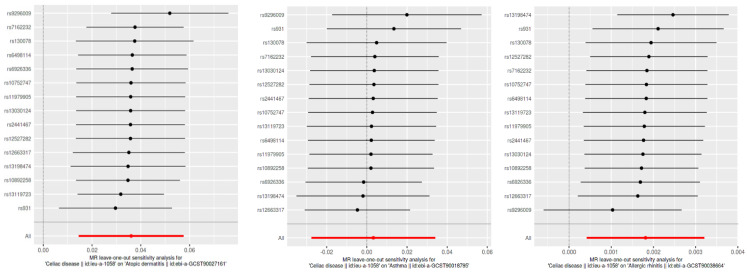
Leave-one-out sensitivity analysis for SNP effects on atopic dermatitis, asthma, and allergic rhinitis. This figure presents the results of the leave-one-out sensitivity analysis for the SNP effects on CD in relation to atopic dermatitis, asthma, and allergic rhinitis. Each plot shows the effect sizes of individual SNPs when one SNP is removed at a time, with the red line representing the overall effect estimate using all SNPs. The analysis ensures that no single SNP disproportionately influences the overall effect, confirming the robustness of our results.

**Figure 5:**
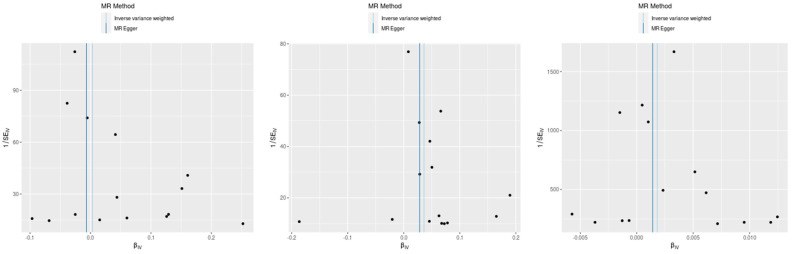
Funnel plots for assessment of publication bias in MR analyses. This figure displays funnel plots for the assessment of publication bias in the MR analyses of the SNP effects on CD with atopic dermatitis, asthma, and allergic rhinitis (**left**: celiac disease and asthma, **center**: CD and atopic dermatitis, and **right**: CD and allergic rhinitis). Each plot shows the relationship between the estimated effect sizes (β) and their standard errors (SE). The vertical line represents the overall effect estimate. The symmetry around this line suggests the absence of publication bias, indicating that the results are not disproportionately influenced by small-study effects or selective reporting.

Causal directionality testing using the MR Steiger approach confirmed that genetic variants associated with celiac disease precede type 2 inflammatory diseases, reinforcing the causality (Steiger *p*-value effectively zero for all outcomes). The sensitivity analyses, including leave-one-out and publication bias assessments, showed stable and unbiased results. However, the practical significance of these findings is limited as the effects fall within the ROPE range, indicating negligible clinical relevance (Figure 4 and Figure 5).

## 4. Discussion 

In our TSMR study, we explored the genetic links between CD and three type 2 inflammatory diseases: atopic dermatitis, asthma, and IgE-mediated food allergies and allergic rhinitis. We found statistically significant associations—an increased risk for atopic dermatitis and allergic rhinitis, a protective effect against asthma, and a non-significant association with IgE-mediated food allergies. However, all observed effects fell within the predefined ROPE range, indicating their lack of clinical significance. This suggests that the hypothesized pathophysiological links between celiac disease and type 2 inflammatory conditions are not substantiated by our data, prompting a reevaluation of their clinical implications. 

Our study uncovers intriguing genetic links between CD and type 2 inflammatory diseases, although the clinical implications of these findings remain limited. The involvement of HLA region variants and regulatory T cells could imply sophisticated genetic interactions influencing the immune responses [16]. However, the clinical relevance of these interactions is not clearly established, limiting their utility in developing therapies based on shared genetic pathways.

The association of specific HLA Class I allelic variations with atopic dermatitis suggests a complex genetic framework that might explain the statistically significant genetic link observed [26,27,28]. Despite this genetic link, the translation of these findings into clinical practice requires additional validation. Similarly, while HLA-DQB1 gene polymorphisms could indicate a relationship with allergic rhinitis [29,30], our data do not confirm a clinically significant connection.

Our findings could align with some evidence in the literature, such as that of Kero et al., who showed that predominantly Th1 and predominantly Th2 diseases can coexist, and Imperatore et al., who found that immune-mediated diseases in celiac patients increase over time regardless of their adherence to a gluten-free diet [13,15]. However, the clinical relevance of these genetic associations, as stated earlier, is still questionable. A possible explanation is the link between SIgAD, associated with CD, and allergic diseases, including type 2 inflammatory conditions [8,9,11]. This dual connection suggests a genetic predisposition. Our TSMR analysis assessed for pleiotropy to ensure the causal relationship between CD and type 2 inflammatory diseases without intermediary connections, but this association could still influence the observed interplay.

One of the key strengths of our study is the use of two-sample Mendelian randomization (TSMR), a method that robustly infers causal relationships and addresses the confounding and reverse causation common in observational studies [18]. Our findings are reinforced by extensive GWAS datasets and a variety of MR techniques, such as IVW, MR Egger, and the weighted median, further supported by sensitivity analyses and the MR Steiger test [31]. Nonetheless, our study’s reliance on data primarily from individuals of European ancestry may limit its generalizability [32]. Additionally, the potential for residual pleiotropy and the exclusion of environmental and lifestyle factors from our analysis could affect the comprehensiveness of our conclusions. Furthermore, the sample size and power of the MR analysis depend on the strength of the genetic instruments; the limited sample sizes for some conditions could reduce the ability to detect true causal effects.

## 5. Conclusions

Our study identifies a tentative genetic connection between CD and type 2 inflammatory diseases, suggesting potential shared immunopathological pathways. Nonetheless, the results, largely falling within the ROPE range, underscore a lack of clinical significance. While these subtle genetic associations may provoke further interest in their underlying biological mechanisms, they currently do not provide a robust basis for the advancement of clinical interventions.

## Data Availability

The data presented in this study were derived from the following resources available in the public domain: GWAS (https://www.ebi.ac.uk/gwas/).
